# 

**Published:** 1894-12-15

**Authors:** 


PRICE TWOPENCE.

No. 429, Vol. XVII.—Dec. 15th, 1894.

Registered at the General Post Office as a Newspaper.]

WITH EXTRA NURSING SUPPLEMENT.

Per Annum, 10s. 6d., post free.

Monthly Parts, 6d.; post free, 8d.

Ditto per Annum, 8s. 6d. post free.

### THE HOSPITAL

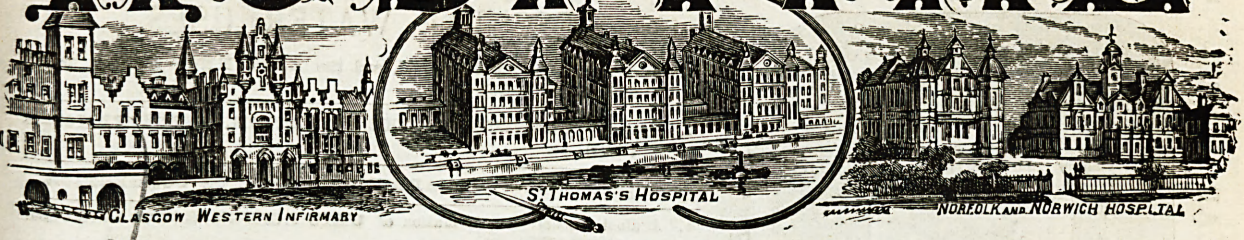

No. 429. Vol. XVII. WITH EXTRA NURSING SUPPLEMENT. SATURDAY, DEC. 15TH, 1894.

